# Evaluation of the Point-of-Care Circulating Cathodic Antigen Assay for Monitoring Mass Drug Administration in a *Schistosoma mansoni* Control Program in Western Kenya

**DOI:** 10.4269/ajtmh.21-0599

**Published:** 2021-11-08

**Authors:** Anne Straily, Emmy A. Kavere, Dollycate Wanja, Ryan E. Wiegand, Susan P. Montgomery, Alex Mwaki, Alie Eleveld, W. Evan Secor, Maurice R. Odiere

**Affiliations:** ^1^ Division of Parasitic Diseases and Malaria, Parasitic Diseases Branch, Centers for Disease Control and Prevention, Atlanta, Georgia;; ^2^ Safe Water and AIDS Project, Kisumu, Kenya;; ^3^ Centre for Global Health Research, Kenya Medical Research Institute, Kisumu, Kenya

## Abstract

The WHO guidelines for monitoring and evaluating *Schistosoma mansoni* control programs are based on the Kato-Katz (KK) fecal examination method; however, there are limitations to its use, particularly in low prevalence areas. The point-of-care urine circulating cathodic antigen (POC-CCA) assay has emerged as a useful tool for mapping schistosomiasis prevalence, but its use in monitoring and evaluating control programs has not been evaluated. Before POC-CCA can be used for these programs, it must be determined how previous guidance based on the KK method can be translated to the POC-CCA assay; furthermore, its performance in different endemicity settings must be evaluated. Urine and stool specimens were collected from students attending public primary schools in western Kenya before mass treatment with praziquantel at baseline (51 schools), year 1 (45 schools), year 2 (34 schools), and year 3 (20 schools). Prevalence and infection intensity were determined by the KK method and POC-CCA assay. Changes in prevalence and intensity were compared within the strata of schools grouped according to the baseline prevalence determined by the KK method (0–10%, > 10–20%, > 20%). The prevalence determined by the POC-CCA assay was higher than that determined by the KK method at all time points for all strata. The prevalence determined by the KK method decreased from baseline to 2 and 3 years, as did infection intensity (with one exception). A corresponding decrease was not always replicated by the POC-CCA assay results. The POC-CCA assay did not perform as expected, and the concordance of results of the two tests was poor. Furthermore, there are emerging concerns regarding the specificity of the POC-CCA assay. Therefore, it is impossible to translate historical data and programmatic guidelines based on the KK method results to the POC-CCA assay.

## INTRODUCTION

Intestinal schistosomiasis, a disease caused by the trematode parasite *Schistosoma mansoni*, is endemic in many sub-Sahara African countries, including Kenya, and affects almost 240 million people globally.^1^ The current WHO-recommended control strategy for schistosomiasis focuses on large-scale treatment of at-risk population groups (mass drug administration [MDA] of praziquantel targeting school-aged children), access to safe water, improved sanitation, hygiene education, and snail control.^2^^,^^3^ The WHO guidelines based the treatment recommendations on the prevalence and intensity of infection among school-aged children because these measures have been determined to be good indicators of the prevalence and infection intensity in the greater community.^4^^–^^8^ Historically, control programs have used the Kato-Katz (KK) testing method^9^ to detect schistosome eggs in stool, make treatment decisions, and monitor program effectiveness, as recommended by the WHO.^10^ However, there are limitations to using the KK method for monitoring and evaluation,^11^ such as its low sensitivity to low-intensity infection levels,^12^^–^^14^ daily variability in egg excretion,^15^^–^^17^ nonhomogenous distribution of eggs in stool,^16^^,^^18^ and requirements of trained laboratory technicians, functioning microscopes, and electricity to perform testing. Although performing repeated KK testing on multiple consecutive stool specimens can increase sensitivity and reduce diagnostic error,^12^ it is logistically impractical for control programs and incurs additional costs.^19^^,^^20^

The point-of-care urine circulating cathodic antigen (POC-CCA) assay is a semi-quantitative test that detects an antigen secreted by the worm and excreted in the urine of the host.^21^^,^^22^ Initial studies indicated that it may be more sensitive than the KK test, especially at lower prevalence levels.^13^^,^^23^^,^^24^ The POC-CCA assay offers additional advantages over the KK method for monitoring control programs. For example, it uses urine specimens, which are less invasive and easier to obtain than stool, there is less variation in the daily excretion levels and between test batches and test readers,^25^ it can be performed in the field, thus foregoing the need to transport specimens back to the laboratory for preparation and analysis, results can be read in 20 minutes, and it is more cost-effective than triplicate KK testing.^19^

In 2013, the WHO published a strategic plan for schistosomiasis control for the years 2012 to 2020, with the goals of controlling schistosomiasis morbidity by 2020 and eliminating schistosomiasis as a public health problem by 2025.^26^ With this strategic plan, the WHO recognized that diagnostic tools used for monitoring and evaluating control programs, such as the KK method, have limitations, and they stated that “there is a need to improve and validate the circulating cathodic antigen assay … for *S. mansoni*.”^26^ Based on the WHO Meeting on Diagnostic Tools for Schistosomiasis Control in February 2015, the Strategic and Technical Advisory Group for Neglected Tropical Diseases encouraged additional validation of the POC-CCA assay to better define cutoffs that should be used for decision-making by *S. mansoni* control programs.

Before the POC-CCA assay can be deployed for the purposes of monitoring and evaluating control programs, it is necessary to determine how to translate previous guidance based on the KK test to the POC-CCA assay and the performance of the test in endemic and post-MDA settings. The overall objective of this study was to determine whether the POC-CCA assay can be substituted for the KK test to monitor the impact and progress of MDA programs. The secondary objectives were to determine the agreement between POC-CCA assay and KK test and the cut-offs for the POC-CCA assay to be used for program decision-making in areas with *S. mansoni* transmission, determine the appropriate frequency of POC-CCA testing for control programs, and determine whether the initial prevalence level among school-aged children based on egg counts affected the usefulness of the POC-CCA assay to monitor the effects of MDA.

## METHODS

### Study design.

In 2017, 30 primary schools were selected from Siaya County (24 schools) and Kisumu County (6 schools). The Kisumu County schools were dropped from the study in 2018, and an additional 21 schools in Siaya County were added, bringing the total number of participating schools to 45; this number remained consistent through 2019. In 2020, sampling was only possible at 30 of the 45 schools before operations were suspended because of the COVID-19 pandemic. Siaya County, which borders Lake Victoria in western Kenya, was selected as the study site because it is an endemic area for intestinal schistosomiasis and has undergone multiple rounds of MDA with praziquantel conducted through the National School Based Deworming Program (NSBDP). In addition, previous studies in the area demonstrated an inverse relationship between schistosomiasis prevalence and distance from the lake,^27^^,^^28^ thus allowing the selection of schools based on the anticipated baseline prevalence. Five of the additional schools in Siaya County were purposively selected because they are a greater distance from the lake (> 10 km) than the other 40 schools; therefore, they were presumed to be approaching an *S. mansoni* prevalence level of 0%. A repeated, cross-sectional design was used throughout the study, and a new random sample of students in selected schools was selected each year. Children aged 9 to 12 years (an approximately equal numbers of males and females) from schools with ongoing MDA provided by the NSBDP were invited to participate. Schools were selected to represent a range of schistosomiasis prevalence levels (0 to < 10%, ≥ 10 to < 20%, and ≥ 20%). The target sample size for each school was 50 to 100 students; students were selected randomly from the school enrollment lists obtained from the headteacher of each school. The sample size of students per school and the number of schools in each strata were calculated using an alpha of 0.05 and 80% power to determine at least a 15% difference in positive POC-CCA assay rates. Permission to conduct the study was obtained from each school and parental/guardian consent was obtained for each child. Children were excluded from participating if they did not provide assent, if they were visibly ill (for example, vomiting or lethargic, as judged by the study nurse and school health teachers), or if they received anthelmintic treatment outside of the NSBDP during the past year (based on information from the school headteacher).

### Test methodology.

In 2017, a single urine specimen and three consecutive daily stool specimens were collected from each participating child at least 1 week before the annual deworming provided by NSBDP. In 2018, 2019, and 2020, a single stool specimen and one urine specimen were collected before MDA. Stool specimens were analyzed using the Kato-Katz method (41.7-mg template)^9^ to detect *S. mansoni* ova; two slides per stool specimen were prepared in 2017, and four slides were prepared for the single stool samples collected during subsequent years. Urine specimens were tested using the commercially available POC-CCA test (Rapid Medical Diagnostics, Pretoria, South Africa; batch numbers: 170316032 for 2017, 180314027 for 2018 and 2019, and 190301016 for 2020; all test kits were used before their expiration dates and transported and stored as recommended by the manufacturer) according to the manufacturer’s instructions. Briefly, two drops of urine were added to the test cassette and allowed to absorb and develop for 20 minutes; then, the results were read. Results were scored based on the intensity of the test band compared with the control band. Tests with no developed test band were scored as negative. Visible bands were scored as positive, with the results ranging from “trace” if the band was barely visible, +1 if the test band was readily visible but less intense than the control band, +2 if the test band had intensity equal to the control band, and +3 if the test band was more intense than the control band. Tests were considered invalid if the internal control band did not develop. All urine samples were also tested for hematuria using a dipstick (Cybow urine reagent strips; DFI Co. Ltd., Gyeongsangnam-do, Korea) and recorded as positive or negative. Laboratory personnel were blinded to the results of the other tests to reduce bias when interpreting agreement between the two tests. Results of the KK and POC-CCA tests were recorded on paper forms and then entered into an electronic database via smartphone using the Open Data Kit application in 2017 and 2018 and the CommCare App (Dimagi Inc., Cambridge, MA) in 2019 and 2020.

### Analyses.

Data were downloaded as an Excel spreadsheet (Microsoft, Redmond, WA). All statistical analyses were performed using SAS (version 9.4; SAS Institute, Cary, NC). The intensity of infection was determined using the KK test based on eggs per gram (epg) of feces and categorized according to the WHO thresholds as light (1–99 epg), moderate (100–399 epg), and heavy (≥ 400 epg).^10^ School-level prevalence was determined as the number of individuals with positive POC-CCA or KK test results divided by the total number of individuals with positive or negative test results multiplied by 100. Individuals were reported as the proportion in the school-level prevalence strata, defined based on KK test results from the first year the school joined the study, and categorized as low (< 10%), medium (10–< 20%) or high (≥ 20%). Baseline measurements were considered as the year when the school joined the study. Comparisons were performed based on the number of years in the study. Mean POC-CCA test scores were determined by assigning a value of 0 to negative results, 0.5 to trace results, 1 to +1, 2 to +2, and 3 to +3. The change in prevalence determined by the POC-CCA and KK test results, mean epg, and mean POC-CCA test scores between years based on the prevalence strata were examined using a Poisson model and negative binomial model, respectively.^29^ Clustering by school was accounted for with generalized estimating equations using a compound symmetric correlation structure.^30^ The 5% level of significance and two-sided tests were used for all analyses. To determine the agreement between the paired KK and POC-CCA test results, contingency tables were created, and the percentages of concordant and discordant paired results were reported. Previous comparison studies indicated that the intensity of the test band on the POC-CCA test corresponds to egg burden.^12^^,^^13^^,^^31^ The intensity of infection determined by the POC-CCA test was categorized by comparing the visual band intensity with the control line and reported as light (trace and +1 combined), moderate (+2), or heavy (+3). We determined the level of agreement between KK and POC-CCA assay results by identifying the number of concordant results as follows: negative POC-CCA and negative KK; trace and +1 POC-CCA (combined) test band and low-intensity infection (1–99 epg) determined by the KK test; +2 POC-CCA band and moderate-intensity infection (100–399 epg) according to the KK test; and +3 POC-CCA band and high-intensity infection (≥ 400 epg) according to the KK test and reported as a percentage.

### Ethical approval.

The study protocol was reviewed and approved initially by the by the KEMRI Scientific and Ethics Review Unit, Nairobi, Kenya (protocol number KEMRI/SERU/CGHR/043/3279), and later by the Institutional Review Board at Maseno University, Kisumu, Kenya (protocol number MSU/DRPI/MUERC/00538/18). Additional approval was obtained from the Kenyan National Commission for Science, Technology, and Innovation. The protocol was also reviewed by the Office of the Associate Director for Science in the Center for Global Health of the CDC; CDC investigators were not considered to be engaged with human subjects.

## RESULTS

There were 51 schools with baseline data; 45, 34, and 20 schools had 1, 2, and 3 years of follow-up data, respectively. The median numbers of participants providing data per school were 100 (range, 60–108) at baseline, 88 (range, 43–100) at 1-year follow-up, 80 (range, 61–100) for schools with 2 years of follow-up, and 84 (range, 54–101) for schools with 3 years of follow-up. Based on the baseline KK data, there were 19 schools in the low prevalence strata (< 10%), 10 schools in the medium prevalence strata (10–20%), and 22 schools in the high prevalence strata (> 20%).

### Change in prevalence determined by the KK and POC-CCA tests.

School-level prevalence determined by the KK and POC-CCA tests at each time point are summarized in Supplemental Table 1; the mean prevalence determined by the KK and POC-CCA tests for participants in the low, medium, and high prevalence strata schools at each time point are summarized in Table 1. Both school-level (Supplemental Table 1) and strata-level mean prevalence rates (Table 1) determined by the KK test were lower than school-level and strata-level mean prevalence rates determined by the POC-CCA test at all time points. The mean strata-level prevalence determined by the KK tests declined for all prevalence strata over the course of all years during the study, except for the low prevalence strata schools between years 2 and 3 (Table 1). All mean strata-level prevalence rates determined by the KK test decreased significantly from baseline to year 2 and from baseline to year 3 (Table 2). The mean strata-level prevalence determined by the POC-CCA test did not uniformly decrease across years in the study; instead, it followed a mostly undulating pattern with a decrease during one year followed by an increase during the next year (Table 1). For low prevalence strata schools, the mean strata-level prevalence determined by the POC-CCA test was essentially unchanged from baseline to year 2 (prevalence ratio [PR], 0.9; 95% CI, 0.67–1.22; *P* = 0.51). Although a decrease was noted from baseline to year 3, it was not significant (PR, 0.73; 95% CI, 0.45–1.18; *P* = 0.2) (Table 2). Among the medium prevalence strata schools, the mean strata-level prevalence determined by the POC-CCA tests increased significantly from baseline to year 2 (PR, 1.19; 95% CI, 1.09–1.31; *P* < 0.01). When baseline data were compared with year 3 data, the decrease was not significant (PR, 0.83; 95% CI, 0.63–1.09; *P* = 0.17) (Table 2). Among the high prevalence strata schools, the mean strata-level prevalence determined by the POC-CCA test increased from baseline to year 2, but it was nonsignificant (PR, 1.09; 95% CI, 0.97–1.23; *P* = 0.16). When baseline data were compared with year 3 data, a significant decrease was observed (PR, 0.71; 95% CI, 0.58–0.87; *P* < 0.01).

**Table 1:** Numbers, prevalence, and infection intensity determined by the Kato-Katz (KK) and point-of-care urine circulating cathodic antigen (POC-CCA) tests according to the school prevalence strata at baseline and years 1, 2, and 3

		Baseline			1 Year			2 Years			3 Years		
Variable	School prevalence strata	No. of schools	n/N or N of participants	% (CI), mean (CI)	No. of schools	n/N or N of participants	% (CI), mean (CI)	No. of schools	n/N or N of participants	% (CI), mean (CI)	No. of schools	n/N or N of participants	% (CI), mean (CI)
KK prevalence	Low	19	95/1815	5.23 (3.96–6.51)	19	72/1,547	4.65 (3.28–6.03)	13	32/1,096	2.92 (1.65–4.19)	6	15/485	3.09 (0.56–5.63)
	Medium	10	142/910	15.6 (13.3–17.9)	9	54/729	7.41 (3.98–10.84)	7	25/532	4.7 (1.25–8.15)	6	12/458	2.62 (0.51–4.73)
	High	22	837/2,086	40.1 (32.77–47.48)	17	533/1,547	34.45 (21.94–46.97)	14	260/1,202	21.63 (8.71–34.56)	8	39/627	6.22 (3.07–9.37)
POC-CCA prevalence	Low	19	936/1,808	51.77 (44.79–58.75)	19	777/1,544	50.32 (45.41–55.24)	13	527/1,101	47.87 (37.02–58.71)	6	202/508	39.76 (16.72–62.81)
	Medium	10	512/908	56.39 (49.22–63.55)	9	348/727	47.87 (38.1–57.63)	7	358/536	66.8 (57.58–76.0)	6	223/479	46.56 (27.87–65.24)
	High	22	1329/2,067	64.3 (58.62–69.97)	17	952/1,539	61.86 (52.37–71.35)	14	903/1,230	73.41 (63.77–83.06)	8	279/640	43.59 (31.99–55.2)
KK intensity (mean eggs per gram, all participants)	Low	19	1,815	2.9 (1.5–4.2)	19	1,547	4.3 (1.8–6.8)	13	1,096	2.2 (0.7–3.8)	6	485	6.4 (1.5–11.2)
	Medium	10	910	10.8 (6.8–14.8)	9	729	11 (5.1–16.9)	7	532	1.6 (0.6–2.5)	6	458	2.9 (0.1–5.7)
	High	22	2,086	69.5 (57.4–81.6)	17	1,547	76.3 (61.8–90.8)	14	1,202	43.1 (32.3–53.8)	8	627	2.7 (0.9–4.5)
POC-CCA intensity (mean band density, all participants)	Low	19	1,808	0.43 (0.4–0.45)	19	1,544	0.5 (0.47–0.54)	13	1,101	0.41 (0.38–0.45)	6	508	0.28 (0.24–0.32)
	Medium	10	908	0.46 (0.43–0.5)	9	727	0.48 (0.43–0.53)	7	536	0.73 (0.66–0.79)	6	479	0.36 (0.31–0.4)
	High	22	2067	0.71 (0.67–0.74)	17	1539	0.84 (0.79–0.88)	14	1,230	0.94 (0.89–1)	8	640	0.31 (0.27–0.34)

**Table 2:** Changes over time during the study from baseline to 1, 2, and 3 years within each school prevalence strata

School prevalence strata	Comparison	Prevalence				Intensity			
		Kato-Katz		Point-of-care urine circulating cathodic antigen		Kato-Katz		Point-of-care urine circulating cathodic antigen	
		Prevalence ratio (95% CI)	*p*	Prevalence ratio (95% CI)	*p*	Arithmetic mean ratio (95% CI)	*p*	Arithmetic mean ratio (95% CI)	*p*
Low	Baseline to year 1	0.91 (0.64–1.30)	0.62	0.97 (0.82–1.15)	0.74	1.51 (0.67–3.4)	0.32	1.18 (0.92–1.52)	0.20
	Year 1 to year 2	0.64 (0.42–0.96)	0.03	0.93 (0.74–1.16)	0.52	0.51 (0.25–1.04	0.06	0.80 (0.62–1.02)	0.08
	Year 2 to year 3	1.01 (0.58–1.75)	0.98	0.81 (0.49–1.33)	0.41	3.08 (1.60–5.95)	0.0008	0.59 (0.39–0.90)	0.01
	Baseline to year 2	0.58 (0.39–0.87)	< 0.01	0.90 (0.67–1.22)	0.51	0.76 (0.35–1.69)	0.51	0.94 (0.65–1.36)	0.74
	Baseline to year 3	0.59 (0.36–0.97)	0.036	0.73 (0.45–1.18)	0.20	2.36 (0.93–5.95)	0.07	0.56 (0.36–0.87)	0.01
Medium	Baseline to 1 year	0.47 (0.32, 0.70)	< 0.01	0.86 (0.71–1.04)	0.13	0.97 (0.57–1.67)	0.92	1.05 (0.81–1.37)	0.71
	Year 1 to year 2	0.68 (0.40–1.15)	0.15	1.39 (1.15, 1.67)	< 0.01	0.19 (0.11, 0.31)	< 0.0001	1.48 (1.06, 2.07)	0.02
	Year 2 to year 3	0.61 (0.30–1.23)	0.17	0.69 (0.55–0.88)	< 0.01	1.9 (0.88–4.06)	0.10	0.51 (0.37–0.69)	< 0.0001
	Baseline to year 2	0.32 (0.18–0.58)	< 0.01	1.19 (1.09–1.31)	< 0.01	0.18 (0.1–0.34)	< 0.0001	1.56 (1.16–2.10)	0.0036
	Baseline to year 3	0.2 (0.12–0.33)	< 0.0001	0.83 (0.63–1.09)	0.17	0.34 (0.15–0.79)	0.01	0.79 (0.54–1.15)	0.22
High	Baseline to year 1	0.80 (0.67–0.97)	0.02	0.91 (0.78–1.06)	0.22	0.95 (0.76–1.18)	0.63	1.03 (0.79–1.35)	0.83
	Year 1 to year 2	0.65 (0.51–0.84)	< 0.01	1.20 (1.02–1.41)	0.02	0.59 (0.41–0.86)	0.0052	1.15 (0.80–1.66)	0.44
	Year 2 to year 3	0.63 (0.54–0.74)	< 0.0001	0.65 (0.55–0.78)	< 0.0001	0.2 (0.13–0.29)	< 0.0001	0.50 (0.38–0.65)	< 0.0001
	Baseline to year 2	0.53 (0.37–0.75	< 0.01	1.09 (0.97–1.23)	0.16	0.56 (0.41–0.77)	0.0004	1.19 (0.86–1.64)	0.29
	Baseline to year 3	0.33 (0.25–0.44)	< 0.0001	0.71 (0.58–0.87)	< 0.01	0.11 (0.08–0.15)	< 0.0001	0.59 (0.46–0.75)	< 0.0001

### Change in the mean infection intensity determined by the KK and POC-CCA tests.

The strata-level mean epg and mean POC-CCA intensity scores of the prevalence strata at each time point are summarized in Table 1. The strata-level arithmetic mean ratios (AMR) for the measures are summarized in Table 2. The mean epg decreased significantly for medium and high prevalence strata schools from baseline to year 2 (medium prevalence strata schools: AMR, 0.18; 95% CI, 0.1–0.34; *P* < 0.0001; high prevalence strata schools: AMR, 0.56; 95% CI, 0.41–0.77; *P* = 0.0004), and year 3 (medium prevalence strata schools: AMR, 0.34; 95% CI, 0.15–0.79; *P* = 0.01; high prevalence strata schools: AMR, 0.11; 95% CI, 0.08–0.15; *P* < 0.0001). Low prevalence strata schools registered a nonsignificant increase in the mean epg from baseline to year 3 (AMR, 2.36; 95% CI, 0.93–5.95; *P* = 0.07). The change in the mean POC-CCA intensity scores were variable over time (Table 2). From baseline to year 2, the mean POC-CCA intensity scores increased significantly for medium prevalence strata schools (AMR, 1.56; 95% CI, 1.16–2.10; *P* = 0.0036), but they were essentially unchanged for low prevalence (AMR, 0.94; 95% CI, 0.65–1.36; *P* = 0.74) and high prevalence strata schools (AMR, 1.19; 95% CI, 0.86–1.64; *P* = 0.29). The mean POC-CCA intensity scores decreased significantly from baseline to year 3 for low prevalence (AMR, 0.56; 95% CI, 0.36–0.87; *P* = 0.01) and high prevalence strata schools (AMR, 0.59; 95% CI, 0.46–0.75; *P* < 0.0001), but not for medium prevalence strata schools (AMR, 0.79; 95% CI, 0.54–1.15; *P* = 0.22).

### Change in infection intensity levels determined by the KK and POC-CCA tests.

**Figure 1.**
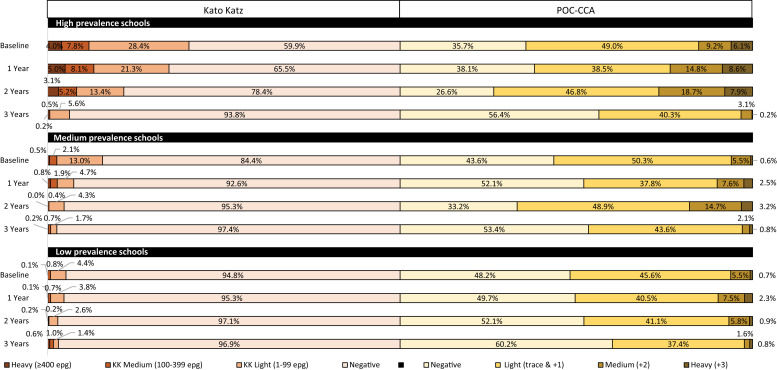
Change in intensity of infections determined by the Kato-Katz (left bars) and point-of-care urine circulating cathodic antigen (POC-CCA) (right bars) tests over time during the study according to the prevalence strata.

Among all prevalence strata, most egg-positive individuals had light-intensity infections (1–99 epg) across all years in the study (Figure 1). Among low prevalence and medium prevalence strata schools, less than 1% of heavy intensity infections (≥ 400 epg) were observed across all years during the study. The percentage of heavy intensity infections determined by the KK test decreased overall for the high prevalence strata schools from 4% at baseline to 3.1% at year 2 and to 0.2% at year 3. Among all prevalence strata, most positive POC-CCA test results were light-intensity infections (trace and +1 combined) (Figure 1). Among low prevalence strata schools at baseline, the percentage of heavy-intensity infections according to the POC-CCA test band (+3) was 0.7% and was virtually unchanged at year 2 (0.9%) and year 3 (0.8%). Among medium prevalence strata schools, the percentage of heavy infections based on the POC-CCA test band intensity increased from 0.6% at baseline to 3.2% at year 2 before decreasing to 0.8% at year 3. Among high prevalence strata schools, the percentage of POC-CCA test heavy intensity bands at baseline was 6.1%; this increased to 7.9% at year 2 and decreased dramatically to 0.2% at year 3. When comparing the percentage of negative POC-CCA test results from baseline to year 3, the percentage increased dramatically for all prevalence strata. However, for schools in the medium prevalence and high prevalence strata, the percentage of negative POC-CCA results decreased from baseline to year 2. Figure 1 depicts these changes over time for each of the prevalence strata.

### Concordance of results of the KK and POC-CCA tests.

Overall, the concordance of positive and negative KK and POC-CCA test results was poor for all prevalence strata at all time points (Tables 3 and 4). Of 12,966 paired specimens collected during the entire study, 5,321 (41%) were negative according to both tests and 1,739 (13.4%) were positive according to both tests. The largest nonconcordance was between egg-negative KK test results and positive POC-CCA test results (5,550; 42.8%). Of the 7,289 positive POC-CCA test results, 1,739 (23.9%) were also positive according to the KK test. When analyzed by year and prevalence strata, the proportions of egg-negative KK test results and positive POC-CCA test results varied considerably (Table 3). There were 356 negative POC-CCA results when eggs were observed using the KK test, representing approximately 2.8% of the total number of paired specimens collected during the study. Most of these false-negative POC-CCA results occurred with infections with light epg intensity determined by the KK test (326; 91.6%). The most false-negative POC-CCA results occurred among high prevalence strata schools (270; 75.8%). Of the 216 high-intensity infections identified by the KK test, only 83 (38.4%) were also categorized as +3 by the POC-CCA test (Table 4); 10 were classified as trace and 40 were classified as +1.

**Table 3:** Summarized discordant positive and negative results of the Kato-Katz (KK) and point-of-care urine circulating cathodic antigen (POC-CCA) tests according to the school baseline prevalence level (determined by KK) strata over time

Discordant test results: negative KK and positive POC-CCA								
	Baseline		1 Year		2 Years		3 Years	
School prevalence strata	Total no. of paired specimens	Discordant pairs, N (%)	Total no. of paired specimens	N (%)	Total no. of paired specimens	N (%)	Total no. of paired specimens	N (%)
Low	1,802	862 (50.4)	1,542	721 (49)	1,093	498 (47)	485	186 (39.6)
Medium	907	396 (51.8)	726	304 (45.2)	528	330 (65.6)	457	204 (45.8)
High	2,062	649 (52.6)	1,536	515 (51)	1,201	641 (68.1)	627	244 (41.5)

Discordant test results: positive KK and negative POC-CCA								
	Baseline		1 Year		2 Years		3 Years	
School prevalence strata	Total no. of paired specimens	Discordant pairs, N (%)	Total no. of paired specimens	N (%)	Total no. of paired specimens	N (%)	Total no. of paired specimens	N (%)
Low	1,802	20 (2.3)	1,542	16 (2.1)	1,093	5 (0.9)	485	4 (1.4)
Medium	907	26 (6.6)	726	10 (2.7)	528	3 (1.7)	457	2 (0.8)
High	2,062	149 (20.3)	1,536	91 (15.5)	1,201	22 (6.8)	627	8 (2.3)

**Table 4:** Concordance of paired Kato-Katz (KK) and point-of-care urine circulating cathodic antigen (POC-CCA) test results for infection intensity

		POC-CCA infection intensity			
		Negative	Low	Moderate	High
KK infection intensity	Negative	48.9%	42.9%	6.6%	1.6%
	Low	22.3%	55.2%	14.5%	7.9%
	Moderate	6.4%	42.2%	30.8%	20.5%
	High	1.4%	23.1%	37.0%	38.4%


Concordance was determined as the number of POC-CCA test results in an infection intensity category according to the number of KK test results in the infection intensity category determined by the KK test from samples collected from the same participant and divided by the total number of KK test results in that infection intensity category. For example, the top left cell is the number of negative POC-CCA test results that were also negative according to the KK test and divided by all the negative KK test samples. The intensity of shading in each cell is based on the percent of concordance. Those cells with higher concordance are shaded darker than those with lower concordance. The POC-CCA test infection intensity categories were defined as low-intensity infections (trace and +1 results combined), moderate-intensity infections (+2 results), and high-intensity infections (+3 results).

## DISCUSSION

Previous studies have demonstrated that when prevalence levels determined by the POC-CCA and KK tests are compared, those determined by the POC-CCA test are almost uniformly higher.^32^ This was true also during our study and was expected; however, the differences in prevalence levels did not vary uniformly among prevalence strata or over time. We anticipated that the positivity rates for both tests would decrease after repeated rounds of MDA. When the KK test was used to assess infection, the prevalence and intensity consistently decreased over time across all strata. However, this was not the case for the POC-CCA test; statistically significant decreases were sporadic, rare, and, during some comparisons, statistically significant increases were observed. CCA levels in urine should decrease rapidly over days to weeks after MDA.^14^^,^^25^^,^^33^ While there is obvious opportunity for reinfection over the course of the yearly intervals used during our study, repeated school-based MDA is still expected to decrease the force of transmission over time, with a resultant decrease in prevalence over time, as was reflected by the KK test results.^34^^–^^36^ Although the sensitivity of the KK test for a single stool can be low,^12^ and we cannot discount the possibility that some infections may have been missed by the KK test, the POC-CCA test results suggested no consistent impact of MDA over time, thus making it an ineffective method of evaluating schistosomiasis control programs. We also did not expect the POC-CCA prevalence to be as high in the known low prevalence schools farther from the lake.

Based on the KK test results in relation to the current WHO guidelines, the schools that participated in this study responded relatively well to MDA, and almost all of the low prevalence and all medium prevalence schools achieved elimination of schistosomiasis as a public health problem (EPHP) (< 1% of heavy-intensity infections) after 2 years.^26^ After examining the infection intensity with the KK test in each individual school, of the 34 schools with at least 2 years of follow-up data, 25 (74%) achieved EPHP during this study (data not shown). Three schools in the high prevalence strata that had not reached the EPHP mark at 2 years did so after an additional year of MDA (data not shown). If similar rules were applied to the POC-CCA test results, and with +3 defined as a heavy-intensity infection, 21 schools (62%) would have achieved EPHP. However, of the nine schools that did not achieve EPHP based on KK test results, four would have achieved EPHP based on the POC-CCA test results. Bärenbold et al. estimated that the prevalence determined by counting only POC-CCA test results ≥ +2 corresponded to the prevalence determined by a single slide evaluated by the KK test^37^; however, when we applied this method to our data, we determined that these measures did not equate well (Figure 1). The lack of concordance between KK test and POC-CCA test results makes translating the previous guidelines based on the KK test difficult because it appears that the relationship between the two tests is not linear.

The frequency of false-negative POC-CCA test results noted during this study is concerning. Previous studies have also reported false-negative POC-CCA results, primarily among light-intensity infections, although most occurred at lower frequencies than identified here. For example, Okoyo et al. (2018) noted false-negative POC-CCA test result rate of 1.3%,^14^ and Lindholz et al. in Brazil noted a similar false-negative rate of 1.7%.^38^ The rate of negative POC-CCA test results that were egg-positive in our study was 2.8%, and the largest rate of false-negative POC-CCA test results occurred among the high prevalence schools. There are several biological explanations to account for the CCA-positive egg-negative results that have been described previously.^39^ To explain the reverse, the manufacturer of the POC-CCA test suggested that very low worm burdens (< 50 worms) may lead to false-negative results.^40^ Sousa et al. reported a 3.2% rate of false-negative POC-CCA test results when comparing the trace positive POC-CCA test results with 16 KK test slides containing three stool specimens in a low- endemic area of Brazil.^41^ Although this frequency was similar to what we observed, most of our false-negative results were observed in a high prevalence area, although they were observed among individuals with light-intensity infections by using the KK test.

The POC-CCA test did not perform as expected. Possible explanations for the unexpected behavior of the POC-CCA test noted during our study could include challenges distinguishing between negative, trace and +1 results determined by the POC-CCA test because most positive POC-CCA test results were in one of the light infection categories. Determining the intensity of the positive test band is subjective, and previous studies have noted the need for better guidelines in this regard.^39^^,^^42^ Another possible explanation that has been proposed is that trace infections may be difficult to clear; potentially representing a few viable adult worms secreting low levels of antigens.^43^^,^^44^ Finally, it must be considered that the current format of the POC-CCA tests may not be sufficiently specific or that some intrinsic error in the test may explain these findings. It has been previously determined that some trace results likely represent false-positive results in low-endemic settings because of inadequate specificity.^39^^,^^43^^,^^45^^,^^46^ Among the five purposively selected schools in a known low-prevalence area, the prevalence determined by the POC-CCA test was much higher than expected, even when accounting for the expected rate of false-positive results. Other recent studies have also begun to identify potential problems with the performance of the test or its specificity in various geographic areas or practice settings. Bezerra et al. compared KK and POC-CCA tests using repeated cross-sectional surveys over the course of 3 years in a low-endemic area of Brazil using a study design similar to ours except that participants were treated with praziquantel only once at baseline.^47^ During that study, all participants had negative CCA results by 6 weeks after treatment; at 1 year, there was only one individual with CCA-positive results; however, at 2 years, the prevalence by POC-CCA had increased to 5.9% when all participants had negative KK test results at all time points, and there was little opportunity for reinfection within the community studied.^47^ In Switzerland, where there is no transmission of schistosomiasis, a group of Eritrean refugees were evaluated at baseline and again 12 to 18 months after treatment. Several individuals had stool microscopy, serology, and POC-CCA test results that were negative at baseline but positive only according to the POC-CCA test during follow-up; the reason for this is difficult to explain.^48^^,^^49^ Furthermore, it was recently documented that there is significant variability between POC-CCA test batches.^50^ A recent study by Graeff-Teixeira et al. demonstrated unacceptably high rates of positive POC-CCA results in a nonendemic area.^51^ In addition, a comparison of communities with long histories of MDA and declining prevalence measured by the KK test demonstrated major discrepancies between two batches of POC-CCA tests, thus leading to concerns about quality control and standardization by the manufacturer.^52^ The POC-CCA tests used during our study were from lots 170316032 (2017), 180314027 (2018 and 2019), and 190301016 (2020); as far as we know, none has been associated with unexpected results in other studies. The higher rate of false-negative POC-CCA test results identified during our study and other recent studies^41^^,^^53^ may indicate that the sensitivity of the test may also be suffering.

This study had some limitations. The study design was a repeat cross-sectional survey; it was not a longitudinal survey. New participants were included each time; therefore, it is unknown if a student tested during a given year actually received MDA during the preceding year, was present during the study during the preceding year, or was a new arrival from an area with differing schistosomiasis endemicity. The variations in sample sizes among students per school and numbers of schools per prevalence strata across time points were also limitations but are likely representative of the situation encountered in schistosomiasis control programs. MDA coverage was not assessed as an effect during this study, but of schools with data regarding MDA coverage, at least 75% achieved target coverage (defined by the WHO as ≥ 75% of school-aged children treated) across all years during the study. Daily fluctuations in both egg output and CCA excretion^15^^–^^17^ can also affect test results; however, with the three consecutive stools (baseline) and subsequent single stools on four slides, we believe daily fluctuations are unlikely to fully explain the outcomes noted. Only a limited number of schools were sampled in 2020 prior to the COVID-19 pandemic; therefore, it is unknown if these schools would have had different responses according to the KK and POC-CCA tests than schools that were not able to be sampled. A strength of this study was that its representativeness is likely robust for children in the area because primary school is compulsory in Kenya. Another strength of this study was that we applied the POC-CCA test in a control program setting, tested its operational applicability as likely would be done in the field, and allowed a direct comparison with the KK test.

In conclusion, we were unable to determine whether the current WHO guidelines for monitoring and evaluating schistosomiasis control programs could be translated from the KK test to the POC-CCA test, or the appropriate testing interval for applying the POC-CCA test. Ignoring that a certain percentage of positive results were false would lead to overtreatment of individuals and communities who may not actually need MDA, thereby incurring increased drug distribution and monitoring costs. Furthermore, if very low worm burdens (a scenario that would also be foreseeable in areas of very low prevalence nearing elimination) or other test errors resulting in a loss of sensitivity in the POC-CCA test would lead to a higher proportion of false-negative results, then the POC-CCA test may not be any better than the traditional KK method. There are many questions surrounding the sensitivity, specificity, and manufacturing practices of the POC-CCA test that need to be answered before it can be used to monitor and evaluate schistosomiasis control programs.

## Supplemental tables


Supplemental materials
