# Supplementary material for: Evaluation of the Point-of-Care Circulating Cathodic Antigen Assay for Monitoring Mass Drug Administration in a Schistosoma mansoni Control Program in Western Kenya
Source: Am J Trop Med Hyg. 2021 Nov 8;106(1):303–11. doi: 10.4269/ajtmh.21-0599 (PMC8733502; doi:10.4269/ajtmh.21-0599)
Supplement: Supplementary file 1 [file tpmd210599.SD1.pdf]

Supplemental Table 1: School-level prevalence determined by KK and POC-CCA at baseline (determined as when the school joined the study), 1, 2, and 3 years.

Prevalence strata	School ID	Baseline		1 Year		2 Years		3 Years	
		KK	POC-CCA	KK	POC-CCA	KK	POC-CCA	KK	POC-CCA
Low	116*	1.0%	52.5%	4.8% ↑	46.4% ↓	n/a	n/a	n/a	n/a
	127*	1.0%	47.5%	6.2% ↑	59.3% ↑	3.4% ↓	71.3% ↑	n/a	n/a
	118*	2.0%	43.0%	8.4% ↑	55.4% ↑	n/a	n/a	n/a	n/a
	124*	3.0%	86.0%	3.5% ↔	57.6% ↓	0.0% ↓	16.7% ↓	n/a	n/a
	113*	3.1%	52.5%	3.4% ↔	50.6% ↓	3.8% ↔	58.8% ↑	n/a	n/a
	132	3.7%	63.0%	4.2% ↔	55.2% ↓	1.3% ↓	63.8% ↑	1.2% ↔	29.5% ↓
	108	4.6%	51.7%	7.4% ↑	50.0% ↓	n/a	n/a	n/a	n/a
	107	4.7%	33.0%	1.1% ↓	45.6% ↑	7.3% ↑	45.9% ↔	2.2% ↓	63.4% ↑
	143	4.8%	42.4%	1.3% ↓	66.2% ↑	0.0% ↓	36.1% ↓	n/a	n/a
	122	5.4%	63.7%	5.5% ↔	30.1% ↓	2.4% ↓	30.1% ↔	n/a	n/a
	137	5.7%	36.8%	3.1% ↓	50.0% ↑	1.0% ↓	62.0% ↑	1.4% ↔	21.8% ↓
	120	5.7%	48.6%	0.0% ↓	25.6% ↓	n/a	n/a	n/a	n/a
	136	6.3%	40.0%	6.7% ↔	62.9% ↑	5.1% ↓	46.5% ↓	n/a	n/a
	146	6.5%	55.1%	8.3% ↑	47.6% ↓	2.5% ↓	68.8% ↑	2.2% ↔	66.0% ↓
	126	7.5%	42.1%	0.0% ↓	53.9% ↑	2.0% ↑	62.0% ↑	4.4% ↑	20.8% ↓
	109	7.6%	62.0%	3.7% ↓	28.0% ↓	n/a	n/a	n/a	n/a
	114	8.5%	67.9%	10.9% ↑	50.0% ↓	6.1% ↓	22.7% ↓	10.0% ↑	30.5% ↑
	121	9.1%	28.4%	8.6% ↔	64.2% ↑	n/a	n/a	n/a	n/a
	147	9.5%	63.7%	2.4% ↓	50.0% ↓	3.0% ↔	28.0% ↓	n/a	n/a
Medium	139	10.8%	53.9%	6.9% ↓	29.2% ↓	4.4% ↓	67.6% ↑	3.0% ↓	49.5% ↓
	145	12.1%	61.3%	8.0% ↓	48.0% ↓	3.7% ↓	59.0% ↑	0.0% ↓	16.0% ↓
	138	13.3%	36.1%	12.0% ↓	51.0% ↑	9.8% ↓	48.5% ↓	2.0% ↓	31.5% ↓
	133	13.3%	31.7%	3.2% ↓	44.4% ↑	n/a	n/a	n/a	n/a
	106	15.1%	60.3%	3.0% ↓	46.3% ↓	9.7% ↑	73.7% ↑	n/a	n/a
	140	17.4%	64.8%	7.1% ↓	37.1% ↓	2.5% ↓	63.3% ↑	2.9% ↔	64.8% ↔
	141	17.4%	55.8%	n/a	n/a	n/a	n/a	n/a	n/a
	117	18.0%	60.0%	14.1% ↓	63.6% ↑	n/a	n/a	n/a	n/a
	151	18.6%	66.0%	1.3% ↓	69.7% ↑	1.6% ↔	78.7% ↑	5.6% ↑	58.2% ↓
	103	19.0%	61.9%	6.1% ↓	36.1% ↓	1.1% ↓	76.1% ↑	1.3% ↔	55.4% ↓
High	129	20.6%	61.3%	9.5% ↓	57.1% ↓	6.1% ↓	60.6% ↑	1.9% ↓	31.6% ↓
	131	21.7%	43.4%	n/a	n/a	n/a	n/a	n/a	n/a
	112	25.0%	48.0%	n/a	n/a	n/a	n/a	n/a	n/a
	115	25.2%	57.0%	10.3% ↓	42.3% ↓	13.9% ↑	80.6% ↑	7.1% ↓	57.6% ↓
	119	27.5%	74.7%	18.5% ↓	48.9% ↓	10.8% ↓	75.7% ↑	3.7% ↓	63.4% ↓
	104	31.0%	41.4%	n/a	n/a	n/a	n/a	n/a	n/a
	102	31.8%	69.6%	8.0% ↓	58.6% ↓	4.4% ↓	57.1% ↓	4.3% ↔	35.7% ↓
	144	35.1%	75.5%	19.1% ↓	51.1% ↓	7.5% ↓	60.2% ↑	6.1% ↓	54.5% ↓
	111	36.1%	69.4%	44.9% ↑	51.0% ↓	36.7% ↓	93.1% ↑	n/a	n/a
	130	36.4%	64.8%	28.3% ↓	63.0% ↓	20.3% ↓	48.8% ↓	11.1% ↓	27.8% ↓
	101	37.4%	46.7%	n/a	n/a	n/a	n/a	n/a	n/a
	105	37.5%	64.6%	16.0% ↓	56.8% ↓	1.6% ↓	63.8% ↑	3.0% ↑	38.0% ↓
	125	40.8%	77.7%	28.0% ↓	25.0% ↓	18.0% ↓	69.0% ↑	12.8% ↓	30.1% ↓
	149	41.6%	50.6%	42.0% ↔	86.0% ↑	18.9% ↓	90.1% ↑	n/a	n/a
	150	42.0%	64.0%	9.1% ↓	61.4% ↑	n/a	n/a	n/a	n/a
	128	42.7%	76.8%	37.0% ↓	58.0% ↓	33.0% ↓	92.9% ↑	n/a	n/a
	135	42.9%	66.7%	40.0% ↓	59.0% ↓	14.0% ↓	68.0% ↑	n/a	n/a
	134	48.0%	71.0%	43.0% ↓	75.6% ↑	15.5% ↓	59.0% ↓	n/a	n/a
	110	51.4%	59.8%	n/a	n/a	n/a	n/a	n/a	n/a
	123	57.7%	69.7%	64.8% ↑	85.7% ↑	n/a	n/a	n/a	n/a
	148	57.8%	73.5%	59.7% ↑	76.4% ↑	n/a	n/a	n/a	n/a
	142	94.9%	92.9%	99.0% ↑	100.0% ↑	92.9% ↓	100.0% ↔	n/a	n/a

↑ = > 1% increase; ↔ = stable within 1%; ↓= > 1% decrease; n/a = no data available (school was not sampled). *Indicates the five purposively selected low prevalence schools that were farther from the lake.
